# Changes in the land-use landscape pattern and ecological network of Xuzhou planning area

**DOI:** 10.1038/s41598-024-59572-9

**Published:** 2024-04-17

**Authors:** Xi Zhou, Zuoyong Chu, Xiang Ji

**Affiliations:** 1 School of Civil Engineering and Architecture, Anhui University of Science and Technology, Huainan, 232001 China; 2 Jiangsu Collaborative Innovation Center for Building Energy Saving and Construction Technology, Jiangsu Vocational Institute of Architectural Technology, Xuzhou, 221000 China

**Keywords:** Landscape pattern and ecological network, Spatiotemporal change, Land-use transition matrix, Landscape pattern indices, Network robustness, Ecology, Environmental sciences, Environmental social sciences, Natural hazards

## Abstract

Ongoing rapid urbanization has triggered significant changes in land use, rendering landscape patterns adversely impacted and certain habitat patches degraded. Ecological networks have consequently contracted overall. As such, an investigation into how land-use landscape patterns and ecological networks change over time and space is of major significance for ecological restoration and regional sustainability. Taking Xuzhou Planning Area as a case study, we examined spatiotemporal changes and features of the landscape pattern by employing the land-use change degree, the land-use transition matrix, and quantified landscape pattern indices. An ecological network analysis, which studies the changes in network connectivity and robustness, as well as their causes and contributors, was undertaken to probe into the features and trends of spatiotemporal changes in the land-use landscape pattern and ecological network amid expeditious urbanization. Analysis results unveiled the following: (1) From 1985 to 2020, there was a decline in the area of farmland, forest, and grassland, accompanied by an increase in land for construction, water bodies, and unused land. The southwestern research area witnessed farmland substantially give way to land for construction for this period, and the most dramatic change in land use occurred between 2000 and 2010. (2) The area of dominant patches in the research area shrank, along with more fragmented, complex landscapes. The land for construction was emerging as the dominant landscape by area, whereas patches of farmland, forest, grassland, and water bodies became less connected. (3) The ecological network was densely linked in the northeast, with sparser connections in the southwest. Spatial shrinkage was observed in the research area’s southwestern and central ecological corridors. Overall, the number of ecological sources and corridors rose and subsequently dropped before a rebound. (4) The ecological network grew more connected and robust from 1985 through 1990, as portions of farmland were converted into water bodies, which led to an increase in ecological sources. Given a reduction in ecological sources and corridors in the southwestern and central regions between 1990 and 2010, network connectivity and robustness declined, which was reversed from 2010 onward with the addition of two ecological sources—Pan’an Lake and Dugong Lake. With an optimal ecological network in 1990, however, it deteriorated significantly by 2010. The research area saw the minimum value of its network connectivity indices of network stability index (α), evenness index (β), and connectivity index (γ), in 2010, when its ecological network was highly fragmented and vulnerable, attributing to a strong contrast between the maximal connected subgraph’s relative size and connectivity robustness. The research findings can lay scientific groundwork for addressing ecological issues, restoring landscape patterns, and developing ecological networks amid urbanization.

## Introduction

Rapid urbanization takes a toll on land use and its resulting landscape pattern, which, in turn, holds back sustainable urban growth^1–4^. In order to safeguard the ecological security of cities and elevate the living standards of their residents, a viable way is to build and improve urban ecological networks^5,6^. An investigation into the features and trends of changes in land use landscape patterns and ecological networks has attracted extensive attention^7–9^.

Presently, a large majority of global research efforts on land use landscape patterns and ecological networks have been made from individual perspectives. On landscape patterns, prior studies focused on examining either the features of spatiotemporal changes concerning all types of the land use landscape through the dynamic degree of land use and land-use transition matrix, metrics derived from land use change^10–15^, or the spatial distribution of land use landscape patterns and features of their spatiotemporal changes by employing landscape pattern indices^16–19^. Nonetheless, there remains a relatively lacking probe into the features of how land use landscape patterns change over time and space from the perspective of ecological networks. In terms of research on ecological networks, much is on the establishment of urban ecological networks comprising ecological sources, corridors, and nodes using morphological spatial pattern analysis (MSPA) and the minimum cumulative resistance (MCR) model^20,21^. Alternatively, the MSPA-InVEST-Conefor model was adopted to single out ecological sources, before building ecological networks and identifying ecological barriers and “pinch points” by leveraging the MCR model and Linkage Mapper, a mapping tool inspired by circuit theory^22–24^, to mark out the to-be-restored areas and further optimize ecological corridors. Overall, the research literature on urban ecological networks primarily has concentrated on how existing corridors are built and refined. However, this line of research is often constrained by a static perspective, overlooking the complex and dynamic nature of various ecological elements, especially lacking consideration of the structural evolution of ecological networks from a long-term time series perspective^25,26^. Internally, ecological networks consist of multiple ecological source areas and corridors. Externally, ecological networks are directly exposed to the surrounding environment, and the uncertainties of external disturbances can cause damage to the networks^27,28^. The extent of this damage is closely related to the topological properties of the ecological networks themselves and changes in landscape elements. Therefore, it is crucial to comprehensively consider network connectivity and robustness over a long time series, quantitatively analyze the changes in urban ecological network topology under external disturbances, and strengthen the comprehensive identification and in-depth study of dynamic evolution characteristics of ecological network structures and critical restoration zones in landscape patterns^29,30^.

Employing the Landsat program’s remote sensing data spanning from 1985 to 2020, we endeavored to elucidate the trends and features of spatiotemporal changes in the landscape pattern and ecological network of the Xuzhou Planning Area. The effort was facilitated by analyzing land-use change and landscape pattern indices and applying ecological network research methods. Commencing with a rigorous exploration of land-use change, this study adopted quantitative measures, including the land-use change degree, the land-use transition matrix, and landscape pattern indices, to discern the intricate spatiotemporal changing trends and characteristics encompassing the urban landscape pattern. Subsequently, the MSPA-InVEST-Conefor model was harnessed to identify ecological sources, before developing an ecological network grounded on the MCR model and under circuit theory. Through the lens of network stability index (α), evenness index (β), and connectivity index (γ)^31^, the ecological network’s connectivity was comprehensively evaluated, and a network simulator with Python was used to create a corresponding complex ecological network model through dual mapping. By subjecting the complex network to stochastic simulated attacks, we explored the network’s robustness in terms of the maximal connected subgraph’s relative size and connectivity robustness. Overall, augmented by an analysis of changes in an ecological network’s connectivity and robustness, the paper illuminates the spatial layout, structural features, and spatiotemporal changes of the landscape pattern and ecological network within the Xuzhou Planning Area for the long time series, aiming to offer constructive insights into urban ecological sustainability, landscape pattern restoration, and ecological corridor optimization.

## Research area overview and data sources

### Research area overview

**Figure 1:**
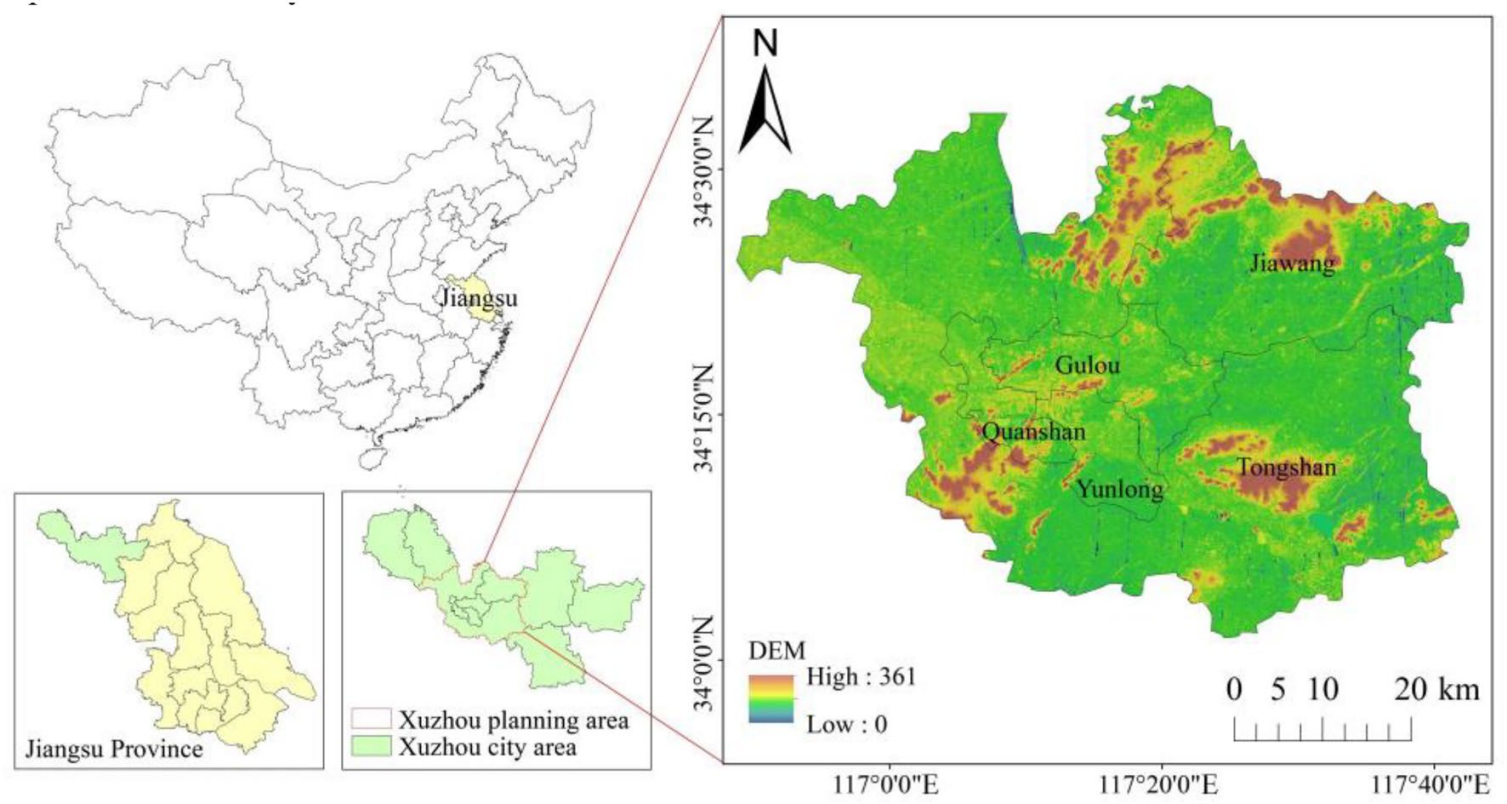
Boundaries of the research areas. (created by ArcMap, version 10.8, http://www.esri.com/).

Situated in the northwest of Jiangsu Province and the southeast of the North China Plain, Xuzhou represents a strategically significant city in the China-proposed Belt and Road Initiative and a national innovation demonstration zone for sustainable development. Plains and hills account for about 90% and 10% of the city’s total area, respectively. The hilly area’s altitude ranges from 100 to 200 m, with the highest peak represented by Dadong Mountain nestled at the heart of Jiawang District, at 361 m. Home to a permanent population of 9.03 million, Xuzhou fosters a robust urbanization rate of 66.19%, and its GDP stood impressively at Renminbi 811.74 billion as of the end of 2021. Factoring in the city’s overarching developmental imperatives and the spatial layout of its landscape pattern and ecological network, we took the Xuzhou Planning Area as our research area, which included downtown Xuzhou and Shuanggou Town in Suining County, as illustrated in Fig. 1. Spanning across 3,062.67 km^2^, the area under research housed 1173 permanent residents per square kilometer by the end of 2021.

### Data sources and processing

The data sources utilized in this research are detailed in Table 1. Upon the geoprocessing of the raw data with the ArcGIS 10.8 software, the corresponding raster data were generated before inputting them in the WGS_1984_UTM_Zone_50N coordinate system*.*

**Table 1:** Remote sensing data sources.

Data type	Data source	Access date
Land-use data	GlobeLand30: http://www.globallandcover.com/	February 11, 2023
Digital elevation model data	Geospatial Data Cloud: https://www.gscloud.cn/	February 11, 2023
Normalized difference vegetation index (NDVI) data	Geospatial Data Cloud: https://www.gscloud.cn/	February 15, 2023
Data on administrative boundaries, highways, and Railways	OpenStreetMap: https://www.openhistoricalmap.org/	February 15, 2023

## Research methods

### Analysis of land-use change

#### Dynamic degree of land use

The dynamic degree of land use reflects the change in the area of land-use types and its intensity for a given time period of research^32,33^. It measures differences between types of land use, time periods, or regions^34^. The equation is specified as follows:

1 $$K = \frac{{U_{{\text{j}}} - U_{i} }}{{U_{i} }} \times \frac{1}{T} \times 100\%$$

where *K* is the dynamic degree index of land use; *U*_*i*_ and *U*_*j*_ are the areas of a particular land-use type in the early and late periods of research, respectively; *T* represents a given time period of research. A larger absolute value of *K* suggests a greater change in land use.

#### Land-use transition matrix

The land-use transition matrix shows information (including about the transitional direction and area) on the mutual conversion of different land-use types during a specific research period (early and late stages)^35,36^. With ArcGIS 10.8, the land-use data of the Xuzhou Planning Area for 1985, 1990, 2000, 2010, and 2020 were rasterized, thus yielding pixel-level data on land-use changes. The data were subsequently processed using Excel, and the land-use transition matrix for the planning area was created. The specific equation is as follows:

2 $$B_{ij} = \left[ {\begin{array}{*{20}c} {B_{11} } & {B_{12} } & \cdots & {B_{1n} } \\ {B_{21} } & {B_{22} } & \cdots & {B_{2n} } \\ \vdots & \vdots & \ddots & \vdots \\ {B_{n1} } & {B_{n2} } & \cdots & {B_{nn} } \\ \end{array} } \right]$$

where *B*_*ij*_ denotes the area of land type *j* converted from land type *i* in the early and late periods of research;* n* is the total number of land-use types.

### Analysis of landscape pattern indices

Landscape pattern indices directly reflect the landscape ecological status of land-use types^37^. In this paper, we selected a variety of indices to evaluate the landscape ecological pattern in a holistic, reasonable manner. At the landscape level, such metrics as the landscape shape index (LSI), area-weighted mean patch fractal dimension (FRAC_AM), contagion index (CONTAG), cohesion index (COHESION), Shannon diversity index (SHDI), Shannon evenness index (SHEI) were preferred^38^. At the patch level, several measures, including class area (CA), number of patches (NP), largest patch index (LPI), LSI, cohesion index (COHESION), and aggregation index (AI)^39^, were chosen. An analysis of the abovementioned indices spanning from 1985 to 2020 allowed us to comprehensively view the ecological status and change of Xuzhou Planning Area’s landscape pattern. Index calculations were conducted with Fragstats 4.2.

### Analysis of ecological network changes

#### Identification of ecological sources

With the land-use data of Xuzhou Planning Area for 1985, 1990, 2000, 2010, and 2020, we considered forest, grassland, and water areas as foreground classes and other land-use types as background classes under MSPA^40^. The eight-neighborhood method was employed to establish seven landscape types, namely cores, islets, perforations, edges, loops, bridges, and branches, of which cores are integral to ecological sources^41^. To determine habitat suitability and factors threatening habitat quality^42,43^, guidance was drawn from such references as the InVEST model user manual and relevant literature^44–46^. Based on this guidance, the weights of factors threatening habitat quality and the habitat sensitivity of all types of land use were produced, as displayed in Tables 2 and 3, respectively. The overall habitat quality of the research area for different time periods was thus measured. Building on this effort, we extracted the 30 largest patches with exceptional habitat quality in the cores and computed their patch importance index (dPC) in landscape connectivity with Conefor 2.6^47^. Patches with a dPC value greater than 1 were designated as ecological sources, while those surpassing a dPC value of 3 were classified as important ecological sources.

**Table 2:** Habitat threat factors and their severity.

Threat factor	Maximum impact Distance	Weight	Spatial degradation type
Farmland	4	0.5	Linear
Land for Construction	10	1	Exponential
Bare Land	2	3	Linear

**Table 3:** Sensitivity of habitat threat factors.

Land-use type	Habitat suitability	Threat Factors		
		farmland	land for Construction	Bare Land
Farmland	0.2	0.3	0.7	0.5
Forest	1	0.7	0.9	0.6
Grassland	0.7	0.6	0.6	0.4
Water bodies	0.9	0.65	0.85	0.5
Land for construction	0	0	0	0
Unused land	0	0	0	0

#### Development of the resistance surface and ecological network

Drawing from relevant literature^48–50^, Six resistance factors, namely habitat quality, land-use types, NDVI, elevation, slope, and topographic relief amplitude, were selected, and weighted under the analytic hierarchy process, as shown in Table 4, to create a comprehensive resistance surface based on the MCR model^51,52^. The circuit theory-inspired Linkage Mapper tool, together with that model, was employed to develop the ecological network of the planning area^53,54^.

**Table 4:** Resistance surface weights and resistance values.

Resistance factor	Grading indicator	Resistance level	Weight	resistance factor	Grading indicator	Resistance level	Weight
Habitat quality	Excellent	1	0.18	Elevation	≤ 30 m	1	0.09
	Above Average	2			30-60 m	2	
	Average	3			60-100 m	3	
	Below Average	4			100-200 m	4	
	Poor	5			≥ 200 m	5	
Land-use type	Forest	1	0.35	Slope	≤ 5°	1	0.08
	Grassland	2			5–10°	2	
	Farmland and Unused Land	3			10–20°	3	
	Water Bodies	4			20–35°	4	
	Land for Construction	5			≥ 35°	5	
NDVI	High Vegetation	1	0.20	Topographic Relief Amplitude	≤ 24°	1	0.08
	Medium–High Vegetation	2			24–44°	2	
	Medium Vegetation	3			44–76°	3	
	Medium–Low Vegetation	4			76–120°	4	
	Sparse Vegetation	5			≥ 120°	5	

### Analysis of structural features of the ecological network

#### Network connectivity

An abstract concept derived from topological space, network connectivity measures the spatial coupling relationship between urban landscape ecological networks and its overall ecological effectiveness^55^. The primary connectivity evaluation indices comprise the alpha index (*α*), which is expressed by the ratio of the actual number of circuits in a network; the beta index (*β*), which reflects the connectivity relating the number of edges to the number of nodes; and the gamma index (*γ*), which measures the connectivity in a network, as presented in Table 5.^56^. By revealing the relationship between ecological nodes and corridor connections, these indices describe the spatial features of urban landscape ecological networks and showcase how complex a network is^57^.

**Table 5:** Network connectivity indices.

Evaluation index	Equation	Description
α	$\alpha = \frac{(L - V + 1)}{{(2V - 5)}}$	0 ≤ α ≤ 1, where α is the number of circuits created by existing nodes. The higher the value, the greater the number of circuits in the network
β	$\beta = \frac{L}{V}$	0 ≤ β ≤ 3, where β describes how difficult a node is connected to another node. The higher the value, the more complex the entire network
γ	$\gamma = \frac{L}{{L_{\max } }} = \frac{L}{{3\left( {V - 2} \right)}}$	0 ≤ γ ≤ 1, where γ reflects the extent to which nodes connect to each other and the resulting ecological effectiveness. The higher the value, the more connected and ecologically effective the network

*V* is the sum of corridor endpoints and points of intersection; *L* represents the number of actual corridors; *L*_*max*_ denotes the maximum possible number of corridors in a network^58^.

#### Network robustness

The robustness of a network is the ability of an ecological network to withstand and survive natural and man-made disasters^59^. Upon direct mapping, the vector graphic of the ecological network of the Xuzhou Planning Area from 1985 to 2020 was converted into a preliminary topological map, which, through remapping, was translated into a corresponding complex network structure chart using the paired method with the Python-enabled NetworkX tool^60^. To assess the robustness of the ecological network, as displayed in Table 6, we investigated how the maximal connected subgraph’s relative size and connectivity robustness perform as the network is under stochastic simulated attacks. The maximal connected subgraph refers to the subset of nodes that remain connected through the fewest edges in the network after some nodes and edges have been compromised due to the attack. Its relative size visually reflects the changes in the topological network structure after damage. Connectivity robustness, on the other hand, signifies the network’s ability to maintain connectivity among its elements and facilitate the transmission of substances and energy even after nodes or edges are dysfunctional due to natural or human-induced disturbances^28,61^.

**Table 6:** Parameters of network robustness evaluation.

Parameter	Equation	Description
Relative size of the maximal connected subgraph	$S = \frac{{C_{\max } }}{N}$	*S* represents the relative size of a maximal connected subgraph; *N* is the number of nodes in the original network; *C*_*max*_ denotes the number of nodes in a maximal connected subgraph
Connectivity robustness	$R = \frac{{C_{\max } }}{{N - N_{r} }}$	*R* implies connectivity robustness; *C*_*max*_ is the number of ecological nodes in a network’s maximal connected subgraph after some nodes are removed; *N* expresses the total number of nodes in a network; *N*_*r*_ denotes the number of nodes removed

## Results and analyses

### Features of spatiotemporal changes in land use

#### Area change and dynamic degree of land use

The areas of land-use types in the Xuzhou Planning Area in 1985, 1990, 2000, 2010, and 2020 and their respective proportions are presented in Table 7. The results showed that from 1985 to 2020, the farmland area gradually decreased, experiencing a reduction of 276.44 km^2^ over 35 years. Both forest and grassland areas displayed an overall declining trend, decreasing by 23.76 km^2^ and 12.91 km^2^, respectively. Concurrently, the areas of land for construction, water bodies, and unused land witnessed an increase, notably the substantial rise in land for construction area, which recorded an increment of 303.80 km^2^ during the 35-year period. The water bodies area expanded and then shrank, followed by an increase in area again, while the unused land area demonstrated a slight rise.

**Table 7:** Areas of land-use types and proportional changes between 1985 and 2020.

Land-Use Type	1985		1990		2000		2010		2020	
	Area	Ratio	Area	Ratio	Area	Ratio	Area	Ratio	Area	Ratio
Farmland	2127.08	71.22	2116.81	70.88	2054.97	68.81	1920.90	64.32	1850.64	61.97
Forest	282.63	9.46	282.80	9.47	279.31	9.35	261.04	8.74	258.87	8.67
Grassland	42.99	1.44	43.17	1.45	42.34	1.42	26.94	0.90	30.08	1.01
Water Bodies	90.49	3.03	98.68	3.30	109.08	3.65	98.27	3.29	99.25	3.32
Land for Construction	439.96	14.73	441.69	14.79	497.45	16.66	675.45	22.62	743.76	25.90
Unused Land	3.45	0.12	3.45	0.12	3.46	0.12	4.02	0.13	4.04	0.14

The units of area and proportion are expressed as km^2^ and %, respectively.

**Figure 2:**
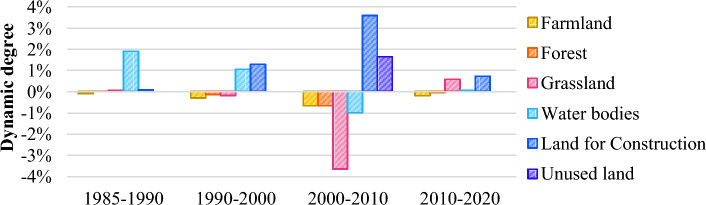
Land-use dynamic degrees between 1985 and 2020.

The dynamic degrees of all land-use types for four specified time periods are illustrated in Figure 2. Between 1985 and 2020, the indicators for land for construction and unused land trended positive. In contrast, those for farmland, forest, and grassland were on a consistently negative trend, and the dynamic degree of water areas was initially positive and subsequently turned negative before returning positive. Land-use changes from 1985 to 1990 were overall modest, with water bodies experiencing the highest dynamic degree of 1.9%. These changes were exacerbated between 1990 and 2000, with land for construction exhibiting the greatest dynamism, at 1.29%. Such changes culminated for the period from 2000 through 2010, in which the dynamic degree of land for construction rose to 3.58%, the highest, while those of farmland, forest, and grassland peaked at -0.65%, -0.65%, and -3.64%, respectively, with that of water areas turning negative. Over the period from 2010 to 2020, land-use changes slowed down, with land for construction showing the highest dynamism, at 0.73%. Overall, the dynamic degrees of all land-use types, except for water bodies, gradually increased from 1985 through 2010 and culminated in the period from 2000 to 2010, followed by a slowdown between 2010 and 2020.

#### Land-use transitions

**Figure 3:**
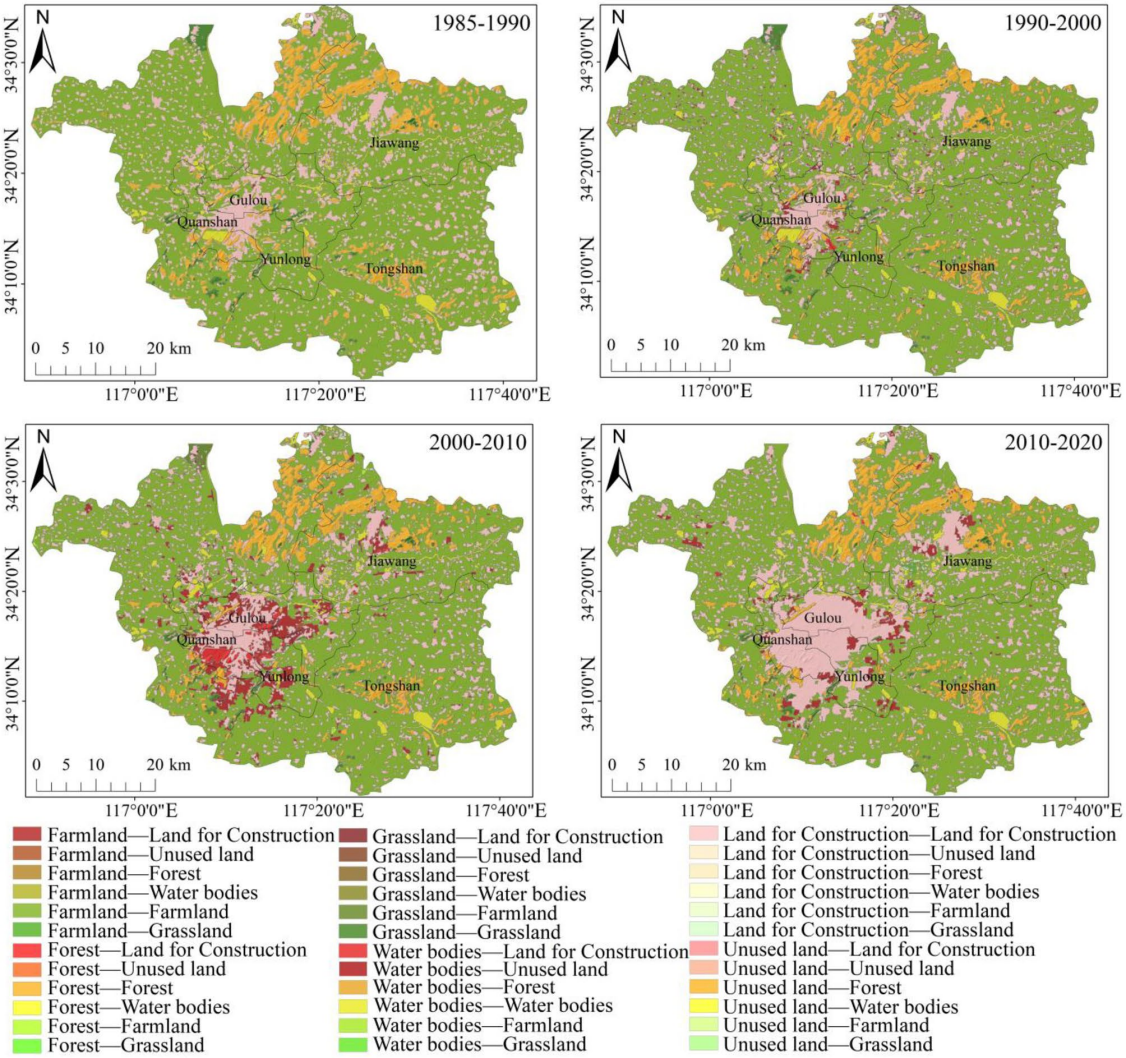
Transitions of land-use types between 1985 and 2020. (created by ArcMap, version 10.8, http://www.esri.com/).

**Figure 4:**
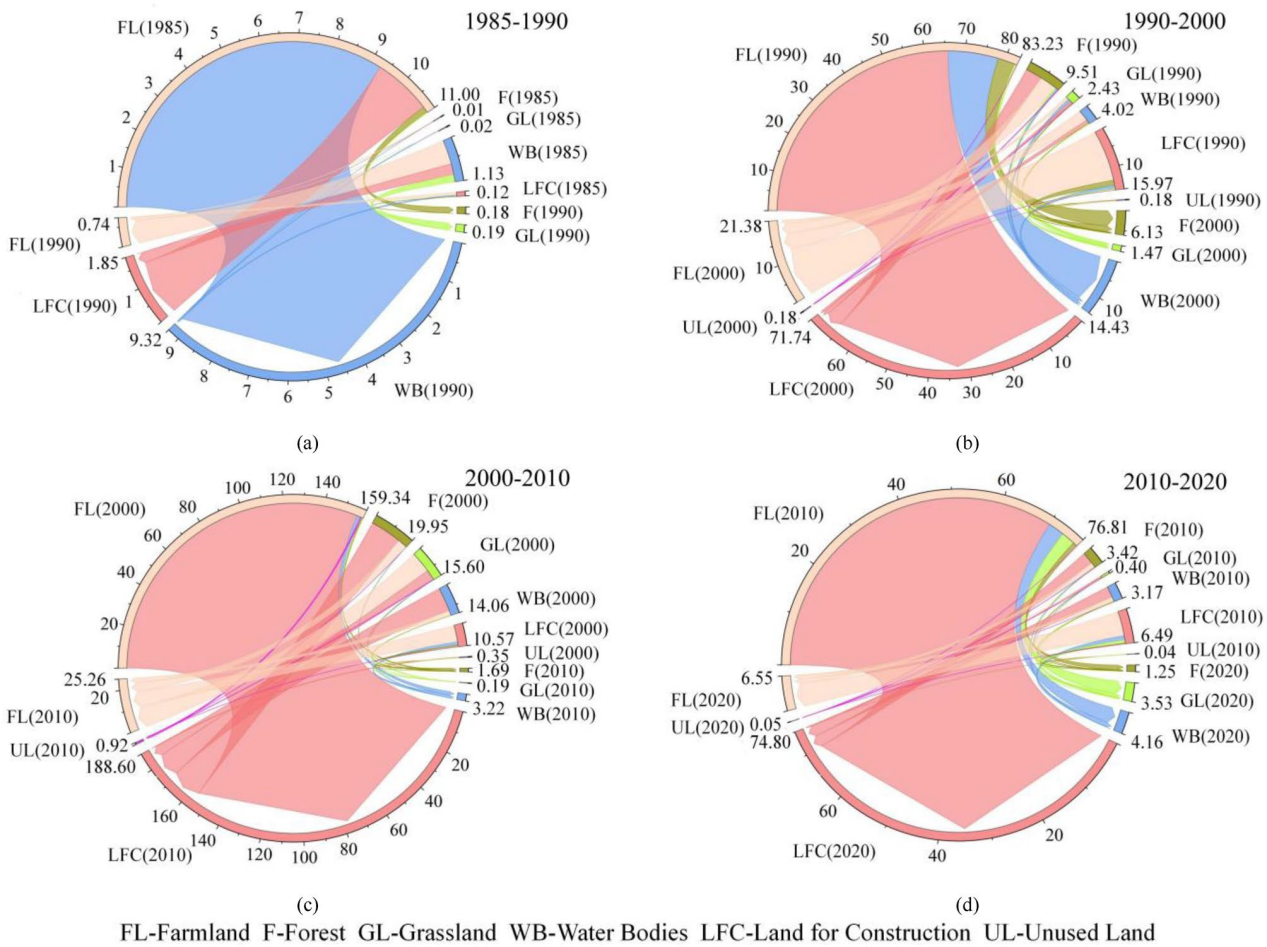
Transitions between different land-use types from 1985 to 2020. (Note: The units in the figure as km^2^.)

In ArcGIS 10.8, maps showing the transitions between land-use types in the research area were generated, as illustrated in Fig. 3, and the corresponding land-use transition matrix was obtained, as displayed in Appendix A (Table A1). Moreover, relevant chordal graphs, as presented in Fig. 4, were created to visually depict transitions between different land-use types. It was observed that: (1) Between 1985 and 1990, land-use transitions primarily involved the conversion of farmland to water bodies and land for construction. The zones where these conversions occurred were dispersed across the research area. (2) From 1990 to 2000, the major land-use transitions took place between farmland and land for construction, with a concentration along the edges of urban areas, particularly in the southwest of the central urban area. Furthermore, other transitions in this period included between farmland and water bodies, between forest and land for construction, and between forest and farmland, which were scattered around various ecological land types. (3) The period from 2000 to 2010 was dominated by the conversion of farmland into land for construction, followed by transitions from forest and water bodies to land for construction, and all these transitions primarily took place in the southwestern central urban area and northeastern Jiawang District. (4) Major transitions between 2010 and 2020 were from farmland to land for construction, mainly concentrated in the southwestern central urban area and the edges of northeastern Jiawang District. Beyond that, land-use transitions could also be seen from land for construction to farmland, farmland to water bodies, farmland to grassland, and forest to land for construction, and notably, conversions from land for construction into farmland occurred around urban built-up areas, while northeastern Jiawang District was dominated by those from farmland into water bodies.

Over the period from 1985 to 2020, a discernible trend of farmland continuously giving way to urban land for construction was observed. Concurrently, farmland progressively diminished, predominantly converting into land for construction; both forest and grassland areas exhibited a declining trajectory, with conversions mainly directed toward farmland and land for construction; the water bodies expanded before contracted, typically reflecting transitions from and to farmland; the area of unused land experienced a consistent increase, chiefly due to transitions from grassland and farmland. The magnitude of land-use transitions between 1985 and 1990 remained relatively modest, with 9.27 km^2^ of farmland transforming into water bodies, resulting in an expansion of water areas. In the subsequent decade (1990–2000), 65.85 km^2^ of farmland was converted into land for construction, concentrated along the periphery of the southwestern central urban area. The most pronounced land-use transitions occurred between 2000 and 2010, during which 156.19 km^2^ of farmland, 15.27 km^2^ of forest, 5.07 km^2^ of grassland, and 11.74 km^2^ of water bodies were made land for construction. The driving force behind such a huge transformation was the sustained expansion of land for construction in the southwestern central urban area, which made surrounding farmland give way. As such, the center of land for construction shifted from the southwest toward the central region of the study area. Rapid urbanization between 2000 and 2010 further converted fragmented farmland in the city outskirts into land for construction and contributed to the reduction in such ecologically valuable land types as forest, grassland, and water bodies. The intensity of land-use transitions from 2010 through 2020 subsided, with 70.02 km^2^ of farmland making room for land for construction. The converted areas were mostly distributed in the southwestern central urban area, northeastern Jiawang District, and the vicinity of fragmented urban areas.

### Features of spatiotemporal changes in the landscape pattern

#### Landscape-oriented evaluation

Variations in landscape-oriented landscape pattern indices in the Xuzhou Planning Area during the period from 1985 to 2020, as depicted in Table 8, offer the following observations. (1) LPI progressively decreased from 70.58 to 60.31, suggesting a trend of diminishing dominant patches and consequential disruption of landscape integrity. Moreover, FRAC_AM exhibited an initial increment, followed by a decline, contributing to an overarching downward trend as its value dropped from 1.2483 to 1.2408. That indicated intensified landscape fragmentation and heightened complexity. (2) CONTAG and COHESION diminished from 58.07 and 99.42 to 54.56 and 99.34, respectively, justifying a multifaceted landscape pattern featured by a decline in overall landscape aggregation and reduced connectivity of dominant patches. (3) SHDI and SHEI surged from 0.92 and 0.51 to 1.02 and 0.57, respectively, indicative of diversifying landscapes and a reduction in landscape evenness. This underscored how dominant landscapes within the research area come to the fore.

**Table 8:** Landscape pattern indices (in the context of landscapes).

Year	LPI	FRAC_AM	CONTAG	COHESION	SHDI	SHEI
1985	70.58	1.2483	58.07	99.42	0.92	0.51
1990	70.26	1.2477	57.73	99.42	0.93	0.52
2000	68.01	1.2485	55.95	99.39	0.96	0.54
2010	62.96	1.2435	55.45	99.37	1.00	0.56
2020	60.31	1.2408	54.56	99.34	1.02	0.57

#### Patch-oriented evaluation

**Figure 5:**
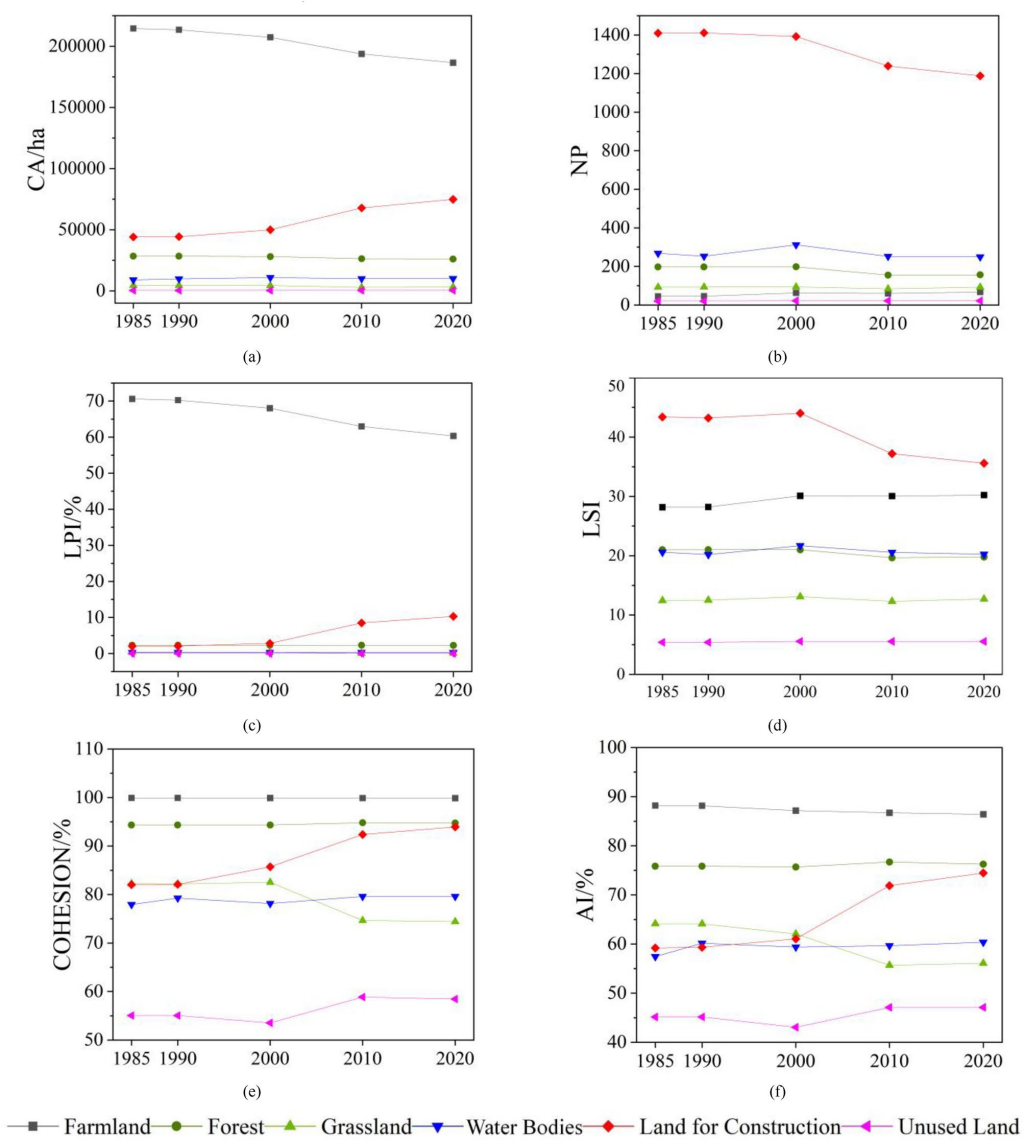
Landscape pattern indices (at the landscape level).

The variations in patch-based landscape pattern indices for the period 1985–2020 in Xuzhou Planning Area are illustrated in Fig. 5, and several notable observations can be made. First, the indices for land for construction, including CA, LPI, COHESION, and AI, were on an upward trend, whereas NP and LSI trended downward, suggesting that patches of land for construction steadily expanded, while becoming less fragmented and more integrated. Its stronger role as the largest dominant landscape type was, to a certain extent, attributed to the process of urbanization. Second, on farmland, such indicators as CA, LPI, COHESION, and AI showed a downward trend, yet its NP and LSI trended upward. That signified a relentless conversion from farmland into land for construction, resulting in a shrinkage in farmland that fragmented the landscape and cut off ties between patches. Third, the six landscape pattern indices for forests between 1985 and 1990 rose, contributing to the expansion and increasing connectivity of forest patches. From 1990 to 2010, its indices of CA, NP, LPI, and LSI dropped, as COHESION and AI went up, which was related to a reduction in patch sizes and complexity within patches. The period from 2010 to 2020 witnessed an increase in the number of patches, despite heightened patch fragmentation and shape complexity, with NP and LSI exhibiting an increase and the remaining indices seeing a fall. Fourth, in terms of grassland, its landscape pattern between 1985 and 2000 varied modestly, but underwent a dramatic change in the following decade (2000–2010), with six landscape pattern indices falling altogether, resulting in a contraction in grassland area and a decentralized, fragmented landscape pattern. Fifth, changes in water areas for the 1985–1990 and 2010–2020 periods were relatively restrained; between 1990 and 2000, the six indices, except for COHESION, rose, which enhanced landscape connectivity as the total area and number of patches increased; despite a rise in AI and COHESION, the readings of CA, NP, LPI, and LSI fell in the subsequent decade (2000–2010), when the total area and number of water bodies sharply decreased amid rapid urbanization. Sixth, a minor change was noted in the landscape pattern of unused land. The number of unused-land patches grew for the period from 1990 to 2000, but dropped in the following decade (2000–2010), which overall indicated a rise in landscape aggregation and complexity followed by a fall.

It is evident from the above analyses that Xuzhou Planning Area is seeing an increasingly fragmented, complex landscape pattern accompanied by decreased landscape connectivity. Specifically, the land for construction is progressively enhancing its role as the largest dominant landscape type, leaving farmland areas decreased and fragmented in the city outskirts. The areas of such ecologically valuable land types as forest, grassland, and water bodies are shrinking, and consequently, patches of these land types are loosely linked. Notably, the period from 2000 to 2010 witnessed an unprecedented urbanization surge, further exacerbating the shrinking area and connectivity of farmland and various ecological land types and amplifying the challenge of landscape fragmentation.In doing so, urban ecological corridors need to be developed and refined as a way of fortifying the city’s ecological network.

### Features of spatiotemporal changes in land use

#### Area change and dynamic degree of land use

**Figure 6:**
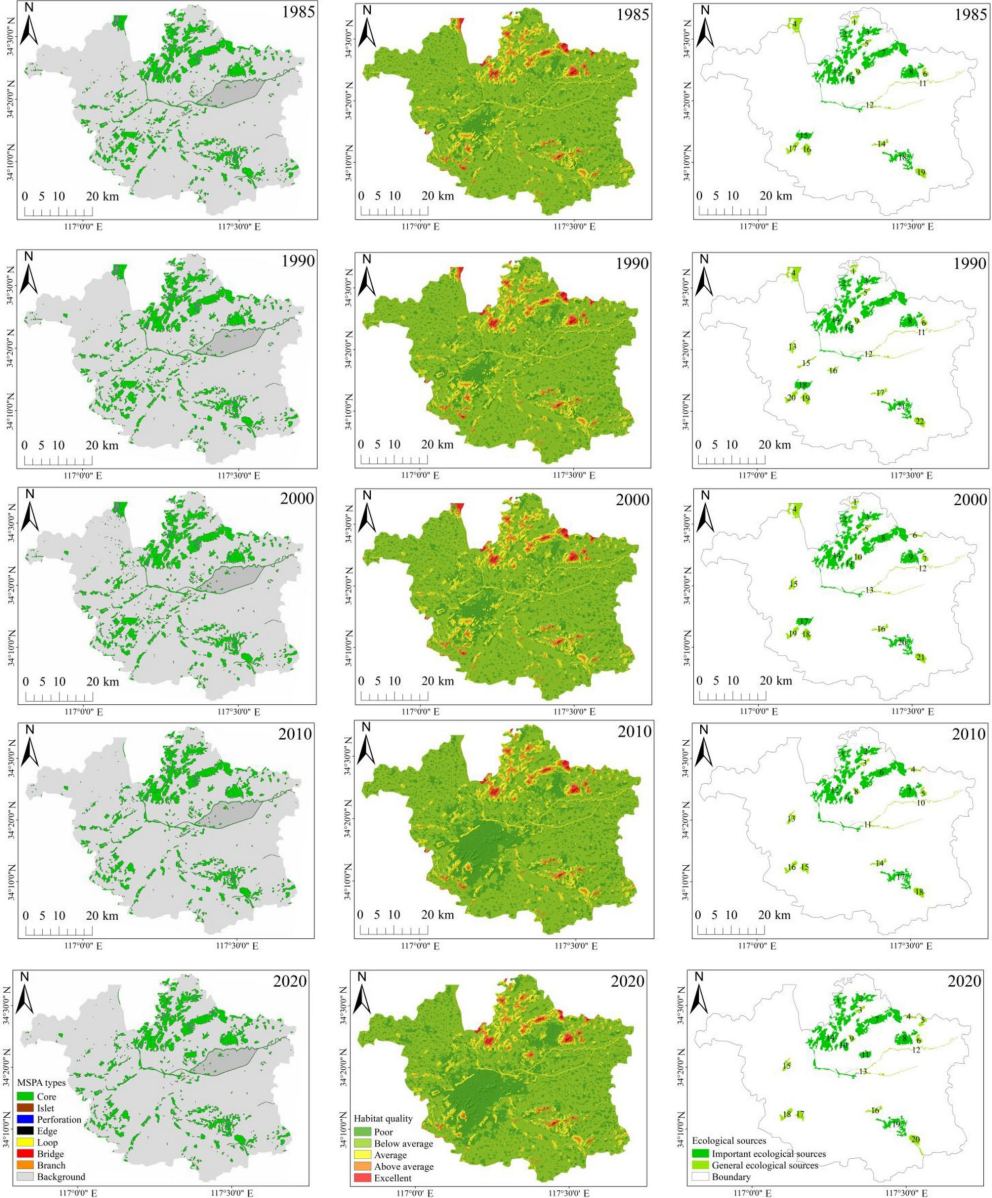
Spatiotemporal changes in ecological sources. (created by ArcMap, version 10.8, http://www.esri.com/).

Based on the MSPA-InVEST analysis outcomes depicted in Figures 6a and b, the top 30 cores with an ecologically valuable habitat in 1985, 1990, 2000, 2010, and 2020 were extracted. And having employed the Conefor 2.6 software for a landscape connectivity analysis, as displayed in Appendix A (Table A2), we identified important and general ecological sources in the Xuzhou Planning Area in each of the five specific years, as illustrated in Fig. 6c.

**Figure 7:**
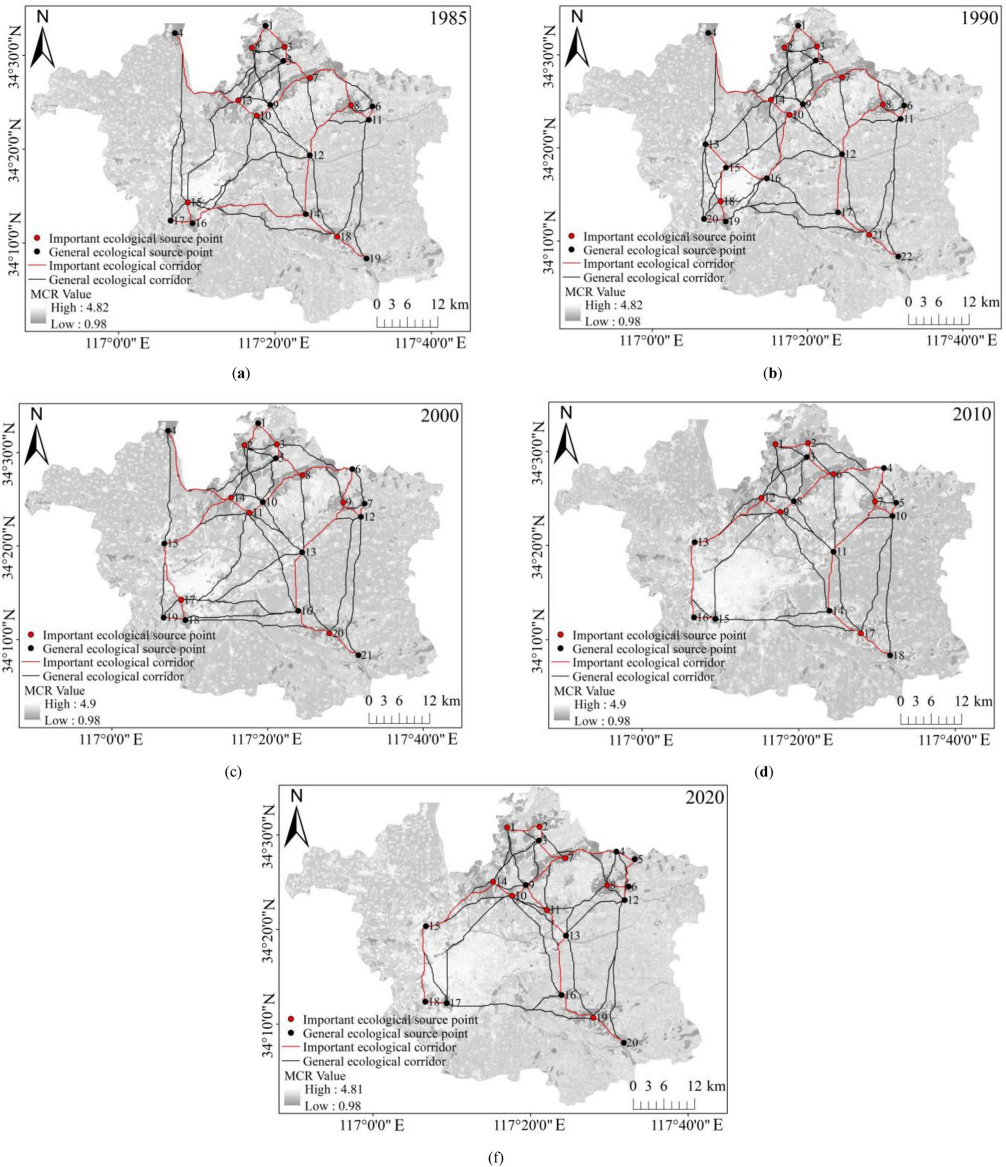
Ecological Corridors. (created by ArcMap, version 10.8, http://www.esri.com/).

**Figure 8:**
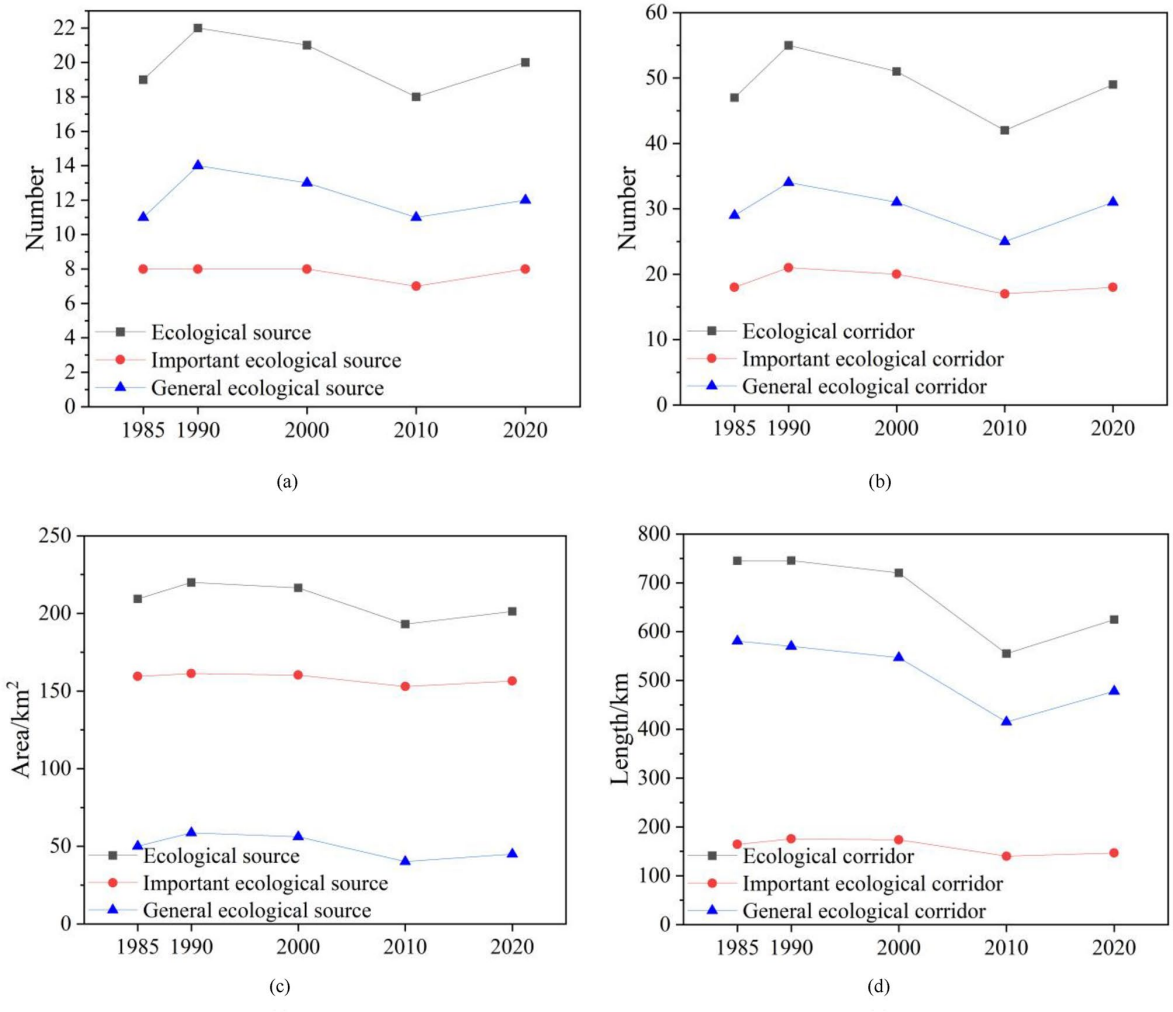
Changes in the ecological-source area and corridor length.

By putting six resistance factors, namely habitat quality, land-use type, NDVI, elevation, slope, and topographic relief amplitude, under overlay analysis, we derived a comprehensive resistance surface for each time period (1985, 1990, 2000, 2010, and 2020). On this basis, the MCR model and the circuit theory-inspired Linkage Mapper tool were leveraged to mark out the important and general ecological corridors in Xuzhou Planning Area in each specific year, as displayed in Figure 7, and to compute how ecological sources and corridors changed over each time period, as shown in Figure 8 and Table 9. Analysis outcomes unveiled that the research area’s ecological corridors were dense in the northeast and sparse in the southwest characterized by intense human activity. Several metrics, such as the area and number of ecological sources and the number and length of corridors, initially increased and subsequently decreased, prior to a final rise. In 1985, a total of 19 ecological source areas were identified, covering a total area of 209.42 km^2^, which accounted for 7.01% of the study area. Based on this, an ecological network comprising 47 corridors was constructed. By 1990, 22 ecological source areas and 55 corridors were identified, with a source area totaling 219.91 km^2^, representing 7.36% of the study area. This period marked the largest source area among the five periods, with corridors exhibiting a relatively uniform distribution. In 2000, due to the development and construction in the southwest central urban area, the ecological resistance in the study area increased, resulting in a reduction of both ecological source areas and corridors in the southwest. Twenty ecological source areas and 51 corridors were identified, reflecting a decrease in source area and corridor length by 3.42 km^2^ and 25.24 km, respectively, compared to 1990. By 2010, rapid urbanization further expanded the southwest central urban area, causing a further reduction in ecological source area and corridor network in the southwest. With 18 ecological source areas and 42 corridors, the proportion of source area in this period was the smallest among the five periods, accounting for only 6.47% of the study area. The ecological network exhibited highly irregular spatial distribution, with a strong presence in the northeast and weak presence in the southwest. In 2020, attention was directed towards ecological restoration. The density of corridors in the central part of the study area increased, with the number of ecological sources rising to 20 and ecological corridors to 49. Overall, there was an increase in the density and importance of ecological corridors in the southwestern research area from 1985 through 1990, with an addition of three ecological sources and eight emerging corridors. Between 1990 and 2000, a reduction of one ecological source and four corridors led to a smaller number of corridors in the southwestern research area. Over the period from 2000 to 2010, an expansion of land for construction in the central urban area further lowered the corridor density within the central research area and shrank the entire corridor area, as evidenced by a decrease of three ecological sources and nine corridors. However, corridors in the northeastern Jiawang District became densely distributed from 2010 through 2020, as the region was joined by another two ecological sources and seven corridors.

**Table 9:** This is a table. Tables should be placed in the main text near to the first time they are cited.

Type	Ecological Source		Important		General		Ecological Corridor		Important		General	
Year	Number	Area	Number	Area	Number	Area	Number	Length	Number	Length	Number	Length
1985	19	209.42	8	159.42	11	50.01	47	745.12	18	164.27	29	580.85
1990	22	219.91	8	161.29	14	58.62	55	745.59	21	175.71	34	569.88
2000	21	216.49	8	160.29	13	56.20	51	720.35	20	173.52	31	546.83
2010	18	193.14	7	152.96	11	40.18	42	555.04	17	140.11	25	414.93
2020	20	201.36	8	156.46	12	44.90	49	624.89	18	146.73	31	478.16

The units of area and Length are expressed as km^2^ and km, respectively.

**Figure 9:**
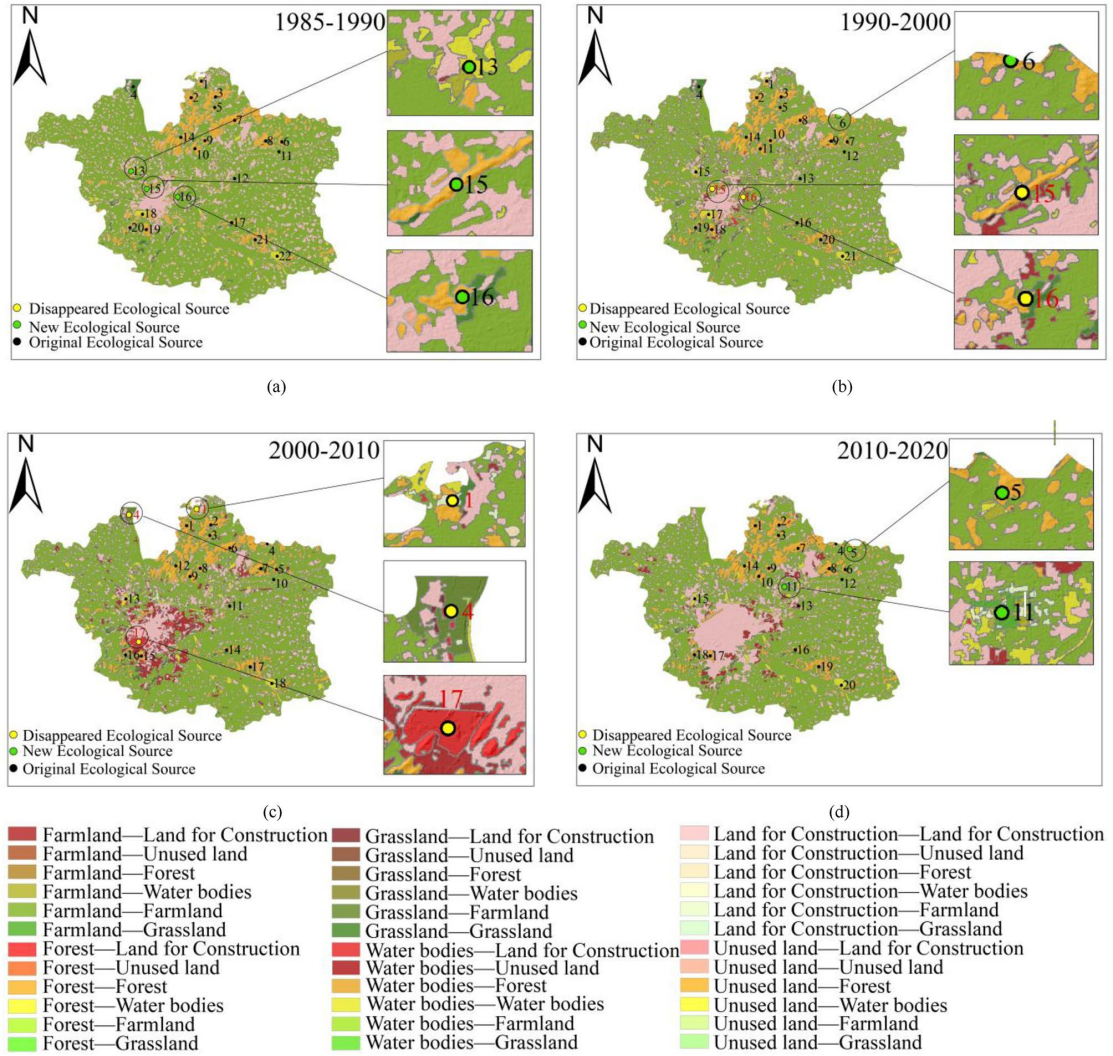
Changes in the ecological-source area and corridor length. (created by ArcMap, version 10.8, http://www.esri.com/).

Upon a deeper analysis that included changes in ecological sources and their corresponding land-use types for each time period, as illustrated in Figure 9, it can be observed that potions of farmland were converted into water areas from 1985 to 1990, with an addition of three ecological sources, namely Jiuli Lake (Dot No. 13), Jiuli Mountain (Dot No. 15), and Baiyun Mountain (Dot No. 16), and eight ecological corridors. This contributed to an increase in the density and importance of corridors in the central research area. Despite that, with an expansion of land for construction in the southwestern urban area between 1990 and 2000, parts of the farmland and forest located in the mountains of Jiuli and Baiyun were used for construction, thus adversely affecting the habitat quality of ecological sources. Besides, certain parcels of farmland in the ecological source of Pengshan Mountain (Dot No. 6), southwest of the research area, were afforested, and with the ecological sources of the mountains of Jiuli and Baiyun disappearing, central and southwestern regions witnessed a decrease in the number of ecological corridors. As the pace of urbanization picked up for the period from 2000 through 2010, the land for construction in the southwest expanded by developing Yunlong Lake (Dot No. 17), certain water bodies of which were utilized for construction purposes. Meanwhile, some parts of grassland and water bodies in the ecological sources of Weishan Lake (Dot No. 1) and Dacheng Mountain (Dot No. 4) made room for built-up areas and farmland, worsening habitat quality and shrinking ecological sources. Beyond that, the central and southwestern research areas witnessed a decrease in the density and distribution range of corridors, with three ecological sources and nine corridors removed. Between 2010 and 2020, with a slowdown in urbanization and efforts made to turn parts of farmland in Dugong Lake (Dot No. 5) and Pan’an Lake (Dot No. 11) back into water bodies, as well as an addition of seven ecological corridors, the density and importance of corridors in northeastern Jiawang District increased.

#### Analyses of changes in structural features of the ecological network

##### Network connectivity analysis

**Figure 10:**
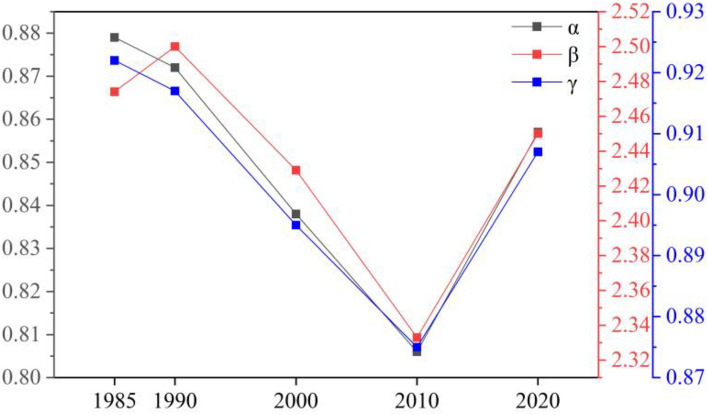
Changes in network connectivity indices.

Variations in network connectivity indices for each particular year of research (namely 1985, 1990, 2000, 2010, and 2020) were presented in Fig. 10 and Table 10. Specifically, the alpha and gamma indices for 1985 peaked throughout the entire research period, with the beta index staying high. That suggested despite being complex, the ecological network was highly connective and connected that year. In 1990, the beta index rose to its highest level between 1985 and 2020, indicative of the most complex network. At the same time, high levels of the alpha and gamma indices justified the heightened connectivity and connectedness of the network. This was attributed to the addition of three ecological sources (Dots No. 13, 15, and 16 in the upper-left map in Fig. 9) and eight corridors, which together strengthened ties between landscapes yet complicated the ecological network. All three indices fell between 1990 and 2010, resulting in a decrease in the ecological index, connectivity, and complexity of the network. The reason was twofold. First, the land for construction expanded in the central urban area under research, leading to the absence of ecological sources and a reduction in ecological corridors and circuits in the southwestern and central regions. Second, a surge in the area of land for construction from 1990 to 2010 increased the ecological resistance value and decreased the number of corridors, rendering the ecological network less connective. In 2010, the alpha, beta, and gamma indices touched their lowest marks, at 0.81, 2.33, and 0.875, respectively. The figures implied that the ecological network was least connected and connective throughout the entire research period, accompanied by a consistent decrease in network complexity. To add to this, the overall ecological network grew dense in the northeast and sparse in the southwest. In 2020, the alpha, beta, and gamma indices rose to 0.86, 2.45, and 0.907, respectively, and the gamma index outperformed that for 2000 and 2010, justifying enhanced network connectivity and connectedness of the ecological network. It apparently was attributed to several factors, including the ecological restoration of the area of coal-mining subsidence at Pan’an Lake in southwestern Jiawang District starting from 2010, the conversion of parts of farmland and land for construction at Jiawang District’s Dugong Lake into water bodies, and the addition of two ecological sources: Pan’an Lake Wetland Park and Dugong Lake. In the same year, the number of ecological corridors and circuits in the central and southwestern research area went up, strengthening the connectivity of the whole ecological network.

**Table 10:** Network connectivity indices.

Year	Alpha Index (α)	Beta Index (β)	Gamma Index (γ)
1985	0.88	2.47	0.922
1990	0.87	2.50	0.917
2000	0.85	2.43	0.895
2010	0.81	2.33	0.875
2020	0.86	2.45	0.907

##### Network robustness analysis

**Figure 11:**
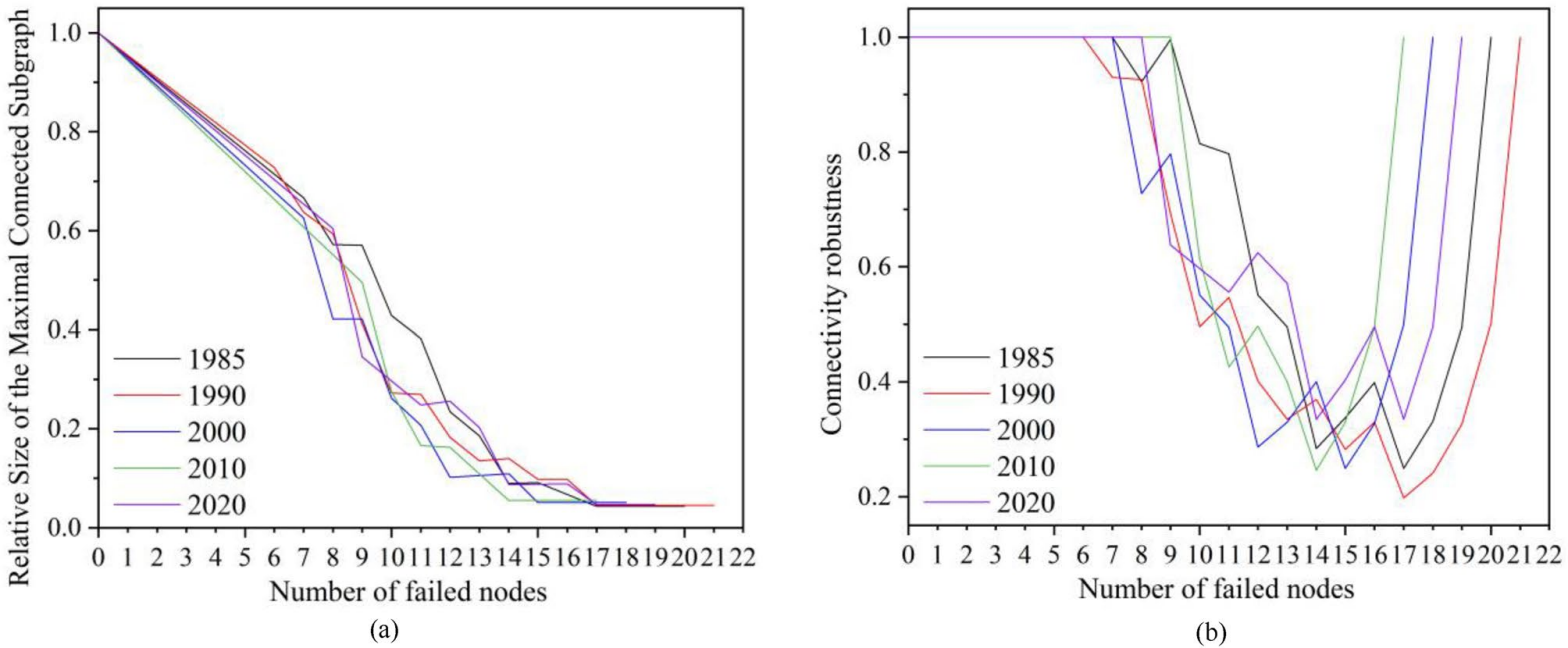
Changes in network connectivity indices. (**a**) Changes in the maximal connected subgraph’s relative size, (**b**) Changes in connectivity robustness.

The prediction of ecological networks in response to natural or human-induced disturbances is of paramount importance in preventing ecological network degradation and biodiversity loss. As random attacks were launched on the ecological network with the Python-enabled Network tool, the changes in the maximal connected subgraph’s relative size and connectivity robustness during the network collapse process were recorded, as shown in Fig. 11. On the relative size of the maximal connected subgraph, it was on a downward trend as the scope of attacks expanded across the ecological network. The decline in the relative size of the maximal connected subgraph accelerated when there were seven failed nodes, and when the figure reached 17, the ecological networks for all five time periods virtually collapsed. Ecological network collapse could be first seen in 2010 when there were 14 failed nodes, indicative of the most dramatic change in the topological structure of the ecological network that year. With the failed node number increasing, the networks for 1985, 2020, 2000, and 1990 broke down in sequence. In 1990, the relative size of the maximal connected subgraph started to fluctuate down when the number of failed nodes increased to six, ending with a network breakdown. Regarding the connectivity robustness of ecological networks, its values over the five time periods under research stood at 1. With an increase in failed nodes, the connectivity robustness of the network trended downward. Such a decline, however, would be reversed as the number of failed nodes reached a certain level that rendered the network structure simpler. It was not until a particular node was attacked that connectivity robustness saw a steep rise, indicative of a changed ecological network structure and a breakdown in cyberspace. When nine nodes failed, the connectivity robustness of the ecological network for 2010 plummeted, and as the number of failed nodes hit 16, its network bore the brunt of a collapse. In 1990, connectivity robustness fluctuated down when nine nodes had a connection problem, but it wound up with a steep rise, which suggested the excellent robustness of the ecological corridor network in this period. The years 1985, 2000, and 2020 witnessed a surge in network connectivity robustness when the numbers of failed nodes reached 17, 18, and 19, respectively. But as the figures continued to rise, connectivity robustness in all three periods was unexceptionally on a decline. It was then established that variations in connectivity robustness and the maximal connected subgraph’s relative size highly aligned with the number of failed nodes. Overall, the ecological network of 1990 exhibited the most robust performance in the face of attacks, followed by the networks of 2000, 2020, 1985, and 2010. The year 2010 saw conspicuous fluctuations and stark differences between the maximal connected subgraph’s relative size and connectivity robustness, indicating a more fragmented ecological network that was subject to heightened vulnerability following disruptions.

## Conclusions and discussions

### Conclusions

Built on multi-source remote sensing imagery for the years 1985, 1990, 2000, 2010, and 2020, the paper investigated the features and trends of spatiotemporal changes in the landscape pattern and ecological network of Xuzhou Planning Area by leveraging the land-use model, landscape pattern indices, and ecological network analysis. Research findings offer guidance for addressing the issue of urban eco-space shrinkage and fragmentation, better preserving ecological resources in the research area, and promoting sustainable urban development. Major conclusions were drawn as follows:

1. Xuzhou Planning Area has undergone a dramatic transformation in the land-use landscape pattern amid urbanization. Between 1985 and 2020, the continuous expansion of land for construction notably encroached upon surrounding farmland, resulting in the conversion of certain ecological land, including forests and grasslands, into construction areas. Over the 35-year period, there was a significant increase in the area of land for construction by 303.08 km^2^, while the areas of farmland, forest, and grassland each decreased by 276.44 km^2^, 23.76 km^2^, and 12.91 km^2^, respectively. The dynamics of water bodies exhibited an initial expansion, followed by a reduction, and then a subsequent increase, while the area of unused land underwent minimal change. Ultimately, the water area and unused land area increased by 8.76 km^2^ and 0.59 km^2^. Urbanization was most pronounced in the period from 2000 to 2010, with land development and construction mainly concentrated in the central urban area of the southwest and Jiawang District of the northeast; From 2010 onward, the pace of urbanization has slowed, and so have changes in land use.
2. Between 1985 and 2020, such landscape-level landscape pattern indices as LPI, FRAC_AM, CONTAG, and COHESION dropped from 70.58, 1.2483, 58.07, and 99.42 to 60.31, 1.2408, 54.56, and 99.34, respectively. The indices of SHDI and SHEI, however, increased throughout the research period, from 0.92 and 0.51 up to 1.02 and 0.57, respectively. These readings suggested that the dominant patch area in landscapes is on a declining trend, accompanied by enhanced ecological spatial heterogeneity and increased fragmentation and complexity of landscapes. At the patch level, the land for construction is steadily enhancing its role as the largest dominant landscape type, and the fragmented invasion of land for construction in the suburbs of cities has caused a reduction and fragmentation of farmland, posing a threat to the quality of habitats and landscape layout such as forests, grasslands, and water bodies.This trend leads to loosely connected patches of these land types.
3. The overall ecological network in the research area grew dense in the northeast and sparse in the southwest characterized by intense human activity. Between 1985 and 2020, the number of ecological sources and corridors rose and subsequently dropped before a rebound. The total area of ecological sources decreased by 8.06 km^2^, despite an increase in number, from 19 to 20. At the same time, the number of corridors rose from 47 to 49, but despite this increase, the combined length was shortened by 120.23 km. Landscapes became better connected for the period from 1985 to 1990, with potions of farmland converted into water areas and an addition of three ecological sources and eight corridors. With an expansion of land for construction in the southwest during the period from 1990 to 2010, the overall number of ecological patches became smaller, thus compromising landscape connectivity and disrupting the ecological landscape pattern. From 2010 onward, landscape connectivity was enhanced as attention was paid to restoring ecosystems, together with a conversion of some farmland portions into water areas and the addition of two ecological sources and seven corridors. On the whole, the northeastern region with dense forest patches had stronger landscape connectivity. The strength of landscape connectivity was correlated with changes in ecological sources, as the expansion of land for construction led to a shrinkage of ecological source areas and corridors in the densely populated southwest, which was unfavorable for material and information exchange and biodiversity conservation in the research area.
4. Over the period from 1985 to 1990, three ecological sources and eight corridors were added, enhancing the connectivity of ecological patches and complicating the ecological network. However, between 1990 and 2010, the spatial distribution of corridors in the southwestern and central research area shrank, resulting in a decline in network connectivity and robustness. Starting from 2010, the addition of two ecological sources, namely Dugong Lake and Pan’an Lake, increased the density of corridors in the central and northeastern research area, thus changing network connectivity and robustness for the better. Overall, the ecological network was most optimal in 1990, with a more even spatial distribution between its source areas, better ensuring the resilience of the ecological network to external disruptions. Followed by 2000, 2020, 1985, and 2010. By 2010, the maximal connected subgraph’s relative size and connectivity robustness exhibited significant fluctuations and differences. And the α, β, and γ indices hit their minimum values of 0.81, 2.33, and 0.875, respectively, indicating that during this period, the period witnessed the most fragmented ecological network, characterized by heightened vulnerability upon disruptions.

### Discussions

In this paper, we delved into land-use landscape patterns changes and transitions in the Xuzhou Planning Area spanning from 1985 through 2020 and produced an ecological network of the research area. Subsequently, we conducted a thorough assessment of network connectivity and resilience, examining the dynamic evolution attributes of the ecological network structure through a prolonged temporal lens and identifying key recovery zones within the landscape schema. Analysis results showed that there were pronounced differences between southwestern and northeastern research regions in terms of the number of ecological corridors and nodes. Another observation was that the ecological network in the northeastern Jiawang District was denser than that in the southwestern research area characterized by intense human activity. This was attributed to the continuous expansion of land for construction in the central urban areas of the southwestern region, with fragmentation encroaching on surrounding farmland and core ecological patches of forest, grassland, and water bodies. Consequently, this has resulted in the contraction of ecological patches and corridors in the southwestern area, propelling the overall landscape towards a trajectory of increased fragmentation and complexity. The inadequate connectivity among patches impedes the exchange of substrates, information, and biodiversity conservation within the study domain. Authorities should avoid allowing uncontrolled construction in these areas if they are to meet the national goal of maintaining habitat connectivity and fostering sustainable urban-rural development. There should be a focus on amplifying ecological restoration and protection initiatives in the southwestern region, rigorously controlling the unplanned expansion and sprawl of construction land, while also intensifying efforts to conserve and connect farmland and core ecological patches of forest, grassland, and water bodies. This includes the establishment of ecological corridors amidst patches, thereby enhancing ecosystem stability and bolstering landscape integration.

Nonetheless, certain limitations in this paper warrant consideration. First, different topographic and geomorphic features in the research area were not factored in during the creation of the complex network, which impeded a further exploration of the interplay between network robustness with ecological nodes under attack and the impact of natural elements in the research area. Second, the processes of extracting ecological sources and weighting ecological resistance factors were primarily drawn from prior research and findings, which entails a qualitative analysis framework. For now, a comprehensive and unified quantitative analytical paradigm for research on ecological networks remains absent. Overcoming these limitations would be instrumental in further complementing the research literature on the structures and features of spatial ecological networks.

### Supplementary Information

Supplementary Information.

## Data Availability

All data generated or analysed during this study are included in this published article [and its supplementary information files].
