# Supplementary material for: Changes in the land-use landscape pattern and ecological network of Xuzhou planning area
Source: Sci Rep. 2024 Apr 17;14:8854. doi: 10.1038/s41598-024-59572-9 (PMC11024202; doi:10.1038/s41598-024-59572-9)
Supplement: Supplementary file 1 — Supplementary Information. [file 41598_2024_59572_MOESM1_ESM.docx]

**Appendix A**

| **Time Period (Year)** | **Land-Use Type** | **Farmland** | **Forest** | **Grassland** | **Water Areas** | **Land for Construction** | **Unused Land** | **Total** |
| --- | --- | --- | --- | --- | --- | --- | --- | --- |
| 1985-1990 | Farmland | 2116.08 | 0.01 | 0.003 | 0.65 | 0.08 | 0 | 2116.81 |
|  | Forest | 0.17 | 282.62 | 0 | 0 | 0.01 | 0 | 282.80 |
|  | Grassland | 0.003 | 0 | 42.98 | 0.19 | 0 | 0 | 43.17 |
|  | Water Bodies | 9.27 | 0.002 | 0.02 | 89.36 | 0.03 | 0 | 98.68 |
|  | Land for Construction | 1.56 | 0.001 | 0 | 0.29 | 439.84 | 0 | 441.69 |
|  | Unused Land | 0 | 0 | 0 | 0 | 0 | 3.45 | 3.45 |
|  | Total | 2127.08 | 282.63 | 42.99 | 90.49 | 439.96 | 3.45 | 2986.60 |
| 1990-2000 | Farmland | 2033.59 | 4.29 | 0.85 | 2.89 | 13.34 | 0.01 | 2054.97 |
|  | Forest | 4.26 | 273.18 | 0.24 | 0.09 | 1.43 | 0.11 | 279.31 |
|  | Grassland | 0.54 | 0.43 | 40.88 | 0.08 | 0.36 | 0.05 | 42.34 |
|  | Water Bodies | 12.55 | 0.37 | 0.69 | 94.65 | 0.82 | 0 | 109.08 |
|  | Land for Construction | 65.85 | 4.32 | 0.61 | 0.96 | 425.70 | 0.01 | 497.45 |
|  | Unused Land | 0.03 | 0.10 | 0.04 | 0 | 0.01 | 3.28 | 3.46 |
|  | Total | 2116.82 | 282.69 | 43.31 | 98.67 | 441.66 | 3.45 | 2986.60 |
| 2000-2010 | Farmland | 1895.64 | 4.56 | 10.28 | 2.28 | 8.13 | 0.01 | 1920.90 |
|  | Forest | 0.66 | 259.36 | 0.07 | 0.01 | 0.93 | 0.01 | 261.04 |
|  | Grassland | 0.06 | 0.10 | 26.75 | 0.02 | 0.01 | 0.003 | 26.94 |
|  | Water Bodies | 1.70 | 0.02 | 0.02 | 95.04 | 1.49 | 0 | 98.27 |
|  | Land for Construction | 156.19 | 15.27 | 5.07 | 11.74 | 486.85 | 0.33 | 675.45 |
|  | Unused Land | 0.73 | 0.02 | 0.17 | 0 | 0.003 | 3.10 | 4.02 |
|  | Total | 2054.97 | 279.31 | 42.35 | 109.10 | 497.43 | 3.45 | 2986.60 |
| 2010-2020 | Farmland | 1844.08 | 0.87 | 0.08 | 0.73 | 4.87 | 0.01 | 1850.64 |
|  | Forest | 0.91 | 257.62 | 0.10 | 0.03 | 0.19 | 0.02 | 258.87 |
|  | Grassland | 2.66 | 0.10 | 26.54 | 0.20 | 0.57 | 0.01 | 30.08 |
|  | Water Bodies | 3.22 | 0.06 | 0.02 | 95.09 | 0.86 | 0 | 99.25 |
|  | Land for Construction | 70.02 | 2.37 | 0.19 | 2.21 | 668.96 | 0.01 | 743.76 |
|  | Unused Land | 0.004 | 0.03 | 0.01 | 0.001 | 0.01 | 3.98 | 4.04 |
|  | Total | 1920.89 | 261.04 | 26.94 | 98.26 | 675.45 | 4.02 | 2986.60 |

**Table A1.** Land-use transition matrix between 1985 and 2020. (Note: The units in the table as km^2^.)

| 1985 | | | 1990 | | | 2000 | | | 2010 | | | 2020 | | |
| --- | --- | --- | --- | --- | --- | --- | --- | --- | --- | --- | --- | --- | --- | --- |
| Source code | DPC | Area  /km^2^ | Source code | DPC | Area  /km^2^ | Source code | DPC | Area  /km^2^ | Source code | DPC | Area  /km^2^ | Source code | DPC | Area  /km^2^ |
| 1 | 1.78 | 3.23 | 1 | 1.75 | 3.23 | 1 | 1.78 | 3.25 | 1 | 6.37 | 9.22 | 1 | 6.11 | 9.22 |
| 2 | 6.47 | 9.23 | 2 | 6.36 | 9.23 | 2 | 6.42 | 9.23 | 2 | 10.84 | 14.29 | 2 | 10.10 | 13.73 |
| 3 | 10.74 | 14.29 | 3 | 10.53 | 14.29 | 3 | 10.65 | 14.29 | 3 | 2.153 | 2.27 | 3 | 2.10 | 2.27 |
| 4 | 2.92 | 10.88 | 4 | 2.908 | 10.88 | 4 | 2.964 | 10.99 | 4 | 1.111 | 2.13 | 4 | 1.11 | 2.12 |
| 5 | 2.11 | 2.28 | 5 | 2.07 | 2.28 | 5 | 2.091 | 2.28 | 5 | 1.50 | 2.93 | 5 | 1.46 | 3.48 |
| 6 | 1.40 | 2.93 | 6 | 1.37 | 2.93 | 6 | 1.05 | 2.13 | 6 | 30.02 | 35.12 | 6 | 1.57 | 2.93 |
| 7 | 28.77 | 35.13 | 7 | 28.20 | 35.13 | 7 | 1.42 | 2.93 | 7 | 9.88 | 16.20 | 7 | 29.24 | 34.26 |
| 8 | 9.45 | 16.49 | 8 | 9.25 | 16.49 | 8 | 28.75 | 35.18 | 8 | 2.45 | 2.45 | 8 | 10.06 | 16.19 |
| 9 | 2.35 | 2.45 | 9 | 2.321 | 2.45 | 9 | 9.56 | 16.49 | 9 | 6.98 | 7.01 | 9 | 2.49 | 2.46 |
| 10 | 6.65 | 6.99 | 10 | 6.60 | 6.99 | 10 | 2.33 | 2.45 | 10 | 1.78 | 3.60 | 10 | 7.06 | 6.98 |
| 11 | 1.68 | 3.60 | 11 | 1.63 | 3.60 | 11 | 6.60 | 6.99 | 11 | 1.63 | 3.66 | 11 | 4.05 | 5.74 |
| 12 | 1.57 | 3.66 | 12 | 1.55 | 3.66 | 12 | 1.68 | 3.60 | 12 | 50.86 | 55.21 | 12 | 1.82 | 3.59 |
| 13 | 47.75 | 53.46 | 13 | 1.09 | 3.42 | 13 | 1.55 | 3.66 | 13 | 1.012 | 3.51 | 13 | 1.65 | 3.45 |
| 14 | 1.13 | 3.82 | 14 | 48.22 | 54.29 | 14 | 47.88 | 53.8 | 14 | 1.16 | 3.81 | 14 | 49.84 | 54.45 |
| 15 | 3.20 | 7.92 | 15 | 1.05 | 2.64 | 15 | 1.26 | 4.01 | 15 | 1.27 | 4.36 | 15 | 1.00 | 3.51 |
| 16 | 2.276 | 5.69 | 16 | 1.01 | 2.55 | 16 | 1.09 | 3.8 | 16 | 1.43 | 5.04 | 16 | 1.19 | 3.82 |
| 17 | 1.87 | 5.05 | 17 | 1.10 | 3.82 | 17 | 3.319 | 8.36 | 17 | 5.22 | 15.87 | 17 | 1.26 | 4.35 |
| 18 | 4.81 | 15.86 | 18 | 3.76 | 8.96 | 18 | 2.16 | 5.60 | 18 | 1.74 | 6.36 | 18 | 1.41 | 5.04 |
| 19 | 1.58 | 6.37 | 19 | 2.33 | 5.69 | 19 | 1.84 | 5.05 |  |  |  | 19 | 5.30 | 15.86 |
|  |  |  | 20 | 1.93 | 5.05 | 20 | 4.55 | 15.86 |  |  |  | 20 | 2.16 | 7.84 |
|  |  |  | 21 | 4.56 | 15.86 | 21 | 1.39 | 6.37 |  |  |  |  |  |  |
|  |  |  | 22 | 1.38 | 6.37 |  |  |  |  |  |  |  |  |  |

**Table A2.** Results of Ecological Patch Landscape Connectivity Calculations from 1985 to 2020.
